# Synthesis and Biological Evaluation of *N*- Pyrazolyl Derivatives and Pyrazolopyrimidine Bearing a Biologically Active Sulfonamide Moiety as Potential Antimicrobial Agent

**DOI:** 10.3390/molecules21091156

**Published:** 2016-08-31

**Authors:** Hend N. Hafez, Abdel-Rhman B.A. El-Gazzar

**Affiliations:** 1 Department of Chemistry, College of Science, Al-Imam Mohammad Ibn Saud Islamic University (IMSIU), P.O. Box: 90950 Riyadh 11623, Saudi Arabia; profelgazzar@yahoo.com; 2 Photochemistry Department, Heterocyclic & Nucleosides Unit, National Research Centre, Dokki, Giza 12622, Egypt

**Keywords:** pyrazole derivatives, pyrazolo[4,3-*d*]pyrimidine, *N*-substituted benzenesulfonamides, antimicrobial agent

## Abstract

A series of novel pyrazole-5-carboxylate containing *N*-triazole derivatives **3**,**4**; different heterocyclic amines **7a**–**b** and **10a**–**b**; pyrazolo[4,3-*d*]pyrimidine containing sulfa drugs **14a**,**b**; and oxypyrazolo[4,3-*d*]pyrimidine derivatives **17**, **19**, **21** has been synthesized. The structure of the newly synthesized compounds was elucidated on the basis of analytical and spectral analyses. All compounds have been screened for their in vitro antimicrobial activity against three gram-positive and gram-negative bacteria as well as three fungi. The results revealed that compounds **14b** and **17** had more potent antibacterial activity against all bacterial strains than reference drug Cefotaxime. Moreover compounds **4**, **7b**, and **12b** showed excellent antifungal activities against *Aspergillus niger* and *Candida albicans* in low inhibitory concentrations but slightly less than the reference drug miconazole against *Aspergillus flavus*.

## 1. Introduction

**Figure 1:**
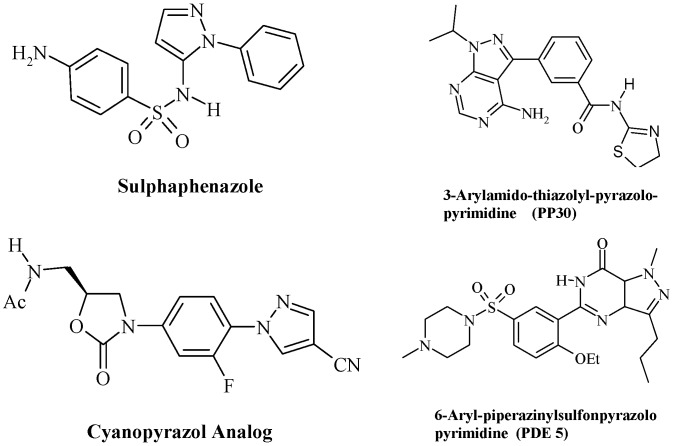
Antimicrobial agents containing pyrazole moiety; pyrazolopyrimidine scaffold based potential candidates and drugs.

Antimicrobial resistance has become a serious health problem, so the increased rate of microbial infections and resistance to antimicrobial agents [1] prompted us to identify a novel structure that may be used in designing new, potent, and broad spectrum antimicrobial agents. Pyrazoles are one of the most common pharmaceutically active compounds, and have attracted much attention due to their broad spectrum of biological activities such as anti-inflammatory [2,3], COX inhibitory [4], hypoglycemic [5], CDK2/Cyclin A [6], p38 MAP kinase [7], and antidepressive activities [8], and have been widely used in biopharmaceutical and pesticides. Pyrazole plays a unique role in drug discovery programs. Pyrazole derivatives are an important class of heterocyclic compounds showing a wide range of biological activities such as antimicrobial [9,10,11] (Figure 1).

Pyrazolopyrimidines have important biological functions including herbicidal [12] and antitumor activity [13]; pyrazolopyrimidine derivatives have been found as purine analogs [14] and have significant properties as antimetabolites in purine biochemical reactions such as neuropeptide Y1 receptor antagonists [15]. Also, the pyrazolo[4,3-*d*]pyrimidinone class of compounds is very important in the treatment of impotence [16], used as an PDE5 inhibitor. PP30 possesses a good kinase selectivity profile used as ATP-competitive mTORC1/mTORC2 inhibitors [17,18] (Figure 1). In view of these observations and as a continuation of our previous work on heterocyclic chemistry, we report herein the synthesis of some new heterocyclic-containing pyrazolo[3,2-*d*]pyrimidine moieties and the study of their antimicrobial activities in comparison to Cefotaxime and miconazole as reference drugs.

## 2. Results

### 2.1. Chemistry

**Scheme 1:**
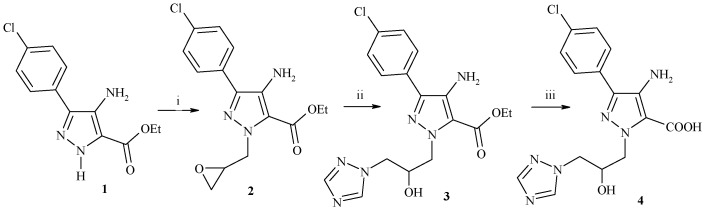
Synthesis of triazolo pyrazole derivatives. *Reagents and conditions:* (i) 2-(chloromethyl)oxirane, K_2_CO_3_, 120 °C, 4 h; (ii) triazole, K_2_CO_3_, EtOH, reflux, 2 h; (iii) 3% NaOH, 100 °C, 3 h.

Pyrazoles are one of the most promising targets for the development of new antimicrobial agents. In our recent work [9], several pyrazole derivatives were designed with potent antibacterial and anticancer activities. In this study, novel small molecules elaborated around *N*-substituted pyrazol-5-carboxylate and pyrazolopyrimidine scaffolds were synthesized. Ethyl 4-amino-3-(4-chlorophenyl)-1*H*-pyrazol-5-carboxylate (**1**) is a precursor to synthesize pyrazole derivatives and pyrazolopyrimidine. Compound (**1**) was synthesized according to the literature [19,20,21,22]. The compound (**1**) was *N*-alkylated by commercial 2-(chloromethyl)oxirane to yield ethyl 4-amino-3-(4-chlorophenyl)-1-(oxiran-2-ylmethyl)-1*H*-pyrazole-5-carboxylate (**2**) in good yield (70%); subsequently, the epoxy ring was opened by 1,2,4-triazole in ethanol using sodium bicarbonate as a base to produce (**3**), and then was further hydrolyzed in water by 3% sodium hydroxide at 100 °C to afford the corresponding 4-amino-3-(4-chlorophenyl)-1-[2-hydroxy-3-(1*H*-1,2,4-triazol-1-yl)-propyl]-1*H*-pyrazole-5-carboxylic acid (**4**) in good yield (Scheme 1).

**Scheme 2:**
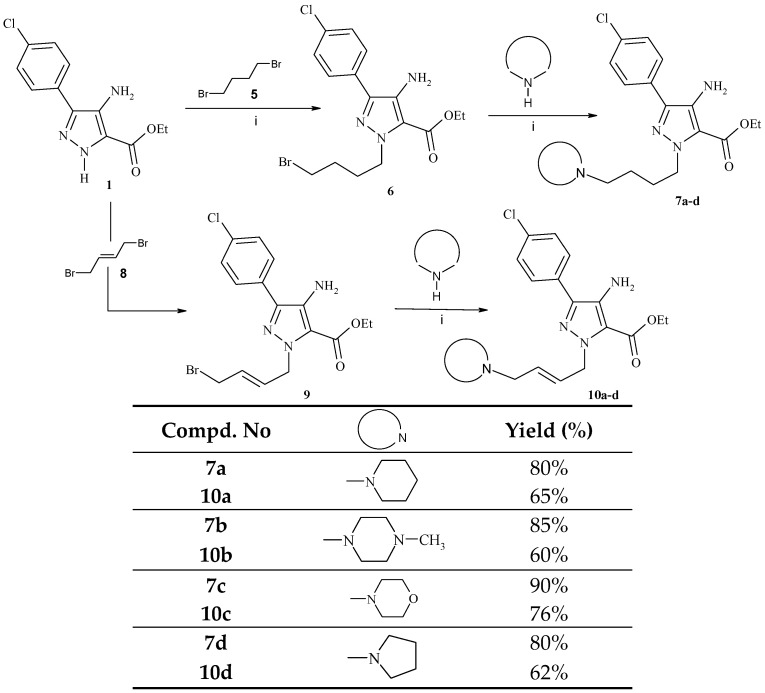
Synthesis of compounds **7a**–**d** and **10a**–**d**; (i) K_2_CO_3_, DMF, 25 °C, 5 h.

The treatment of ethylcarboxylate (**1**) with an excess of 1,4-dibromobutane (**5**) in the presence of K_2_CO_3_ in DMF at 25 °C for 5 h obtained crude ethyl-4-amino-1-(4-bromobutyl)-3-(4-chlorophenyl)-1*H*-pyrazole-5-carboxylate (**6**). Similarly, ethyl 4-amino-1-[(2*E*)-(4-bromobut-2-en-1-yl)-3-(4-chlorophenyl)-1*H*-pyrazole-5-carboxylate (**9**) was prepared from (**1**) and (*E*)-1,4-dibromobut-2-ene (**8**). Compounds (**6**) and (**9**), when reacted separately with different heterocyclic secondary amines, gave the corresponding desired products, (**7a**–**d**) and (**10a**–**d**), respectively, in good yields (Scheme 2).

The treatment of ethyl 4-amino-3-(4-chlorophenyl)-1*H*-pyrazol-5-carboxylate (**1**) with thiophosgene gave the corresponding 3-isothiocyanat derivatives (**11**). The reactivity of isothiocyanate (**11**) towards nitrogen nucleophile was investigated. Thus, interaction of (**11**) with sulfa drugs in dimethylformamide at room temperature yielded the corresponding ethyl-3-(4-chlorophenyl)-(3-(4-(*N*-substitutedsulfamoyl)phenyl)thioureido-1*H*-pyrazole-5-carboxy-late (**12a**–**d**), respectively.

**Scheme 3:**
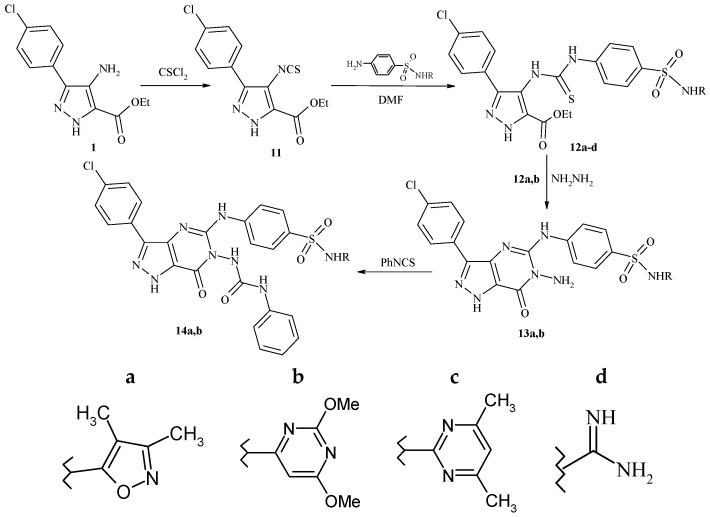
Synthesis of pyrazolo[4,3-*d*]pyrimidin[1,2]isooxazolobenzenesulfonamide (**14a**), benzenesulfonamide (**14b**) via **12a**–**d** and **13a**,**b**.

Treatment of compounds (**12a**,**b**) with hydrazine hydrate in refluxing ethanol afforded the cyclic *N*-amino compounds (**13a**,**b**). The formation of *N*-amino derivatives (**13a**,**b**) proceeded via loss of 1 mol of H_2_S, followed by intramolecular cyclization to give (**13a**,**b**), respectively. IR spectra of compounds (**13a**,**b**) revealed the presence of characteristic bands of NH_2_, C=O and SO_2_ groups. Thioureido derivatives (**14a**,**b**) were obtained via a reaction of compounds (**13a**,**b**) with phenyl isothiocyanate (Scheme 3).

**Scheme 4:**
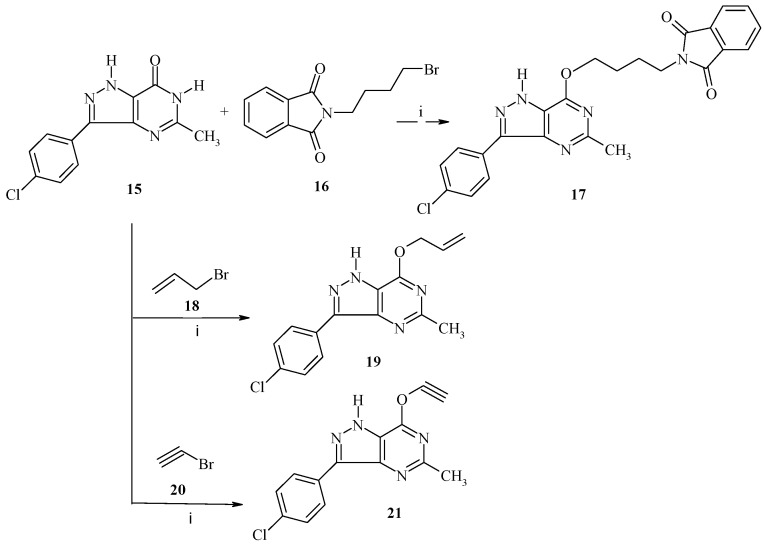
Reaction of 3-(4-chlorophenyl)-5-methyl-1,6-dihydro-7*H*-pyrazolo[4,3-*d*]pyrimidin-7-one (**15**) with 2-(4-chlorobutyl)isoindoline-1,3-dione (**16**), allyl bromide (**18**) and bromoethyne (**20**); (i) K_2_CO_3_, DMF, 25 °C, 10 h.

Compound 3-(4-chlorophenyl)-5-methyl-1,6-dihydro-7*H*-pyrazolo[4,3-*d*]pyrimidine-7-one (**15**) was synthesized according to the literature [2], which on treatment with 2-(4-bromobutyl)-isoindoline-1,3-dione (**16**) in presence of K_2_CO_3_ in DMF at 25 °C for 6 h gave O-substituted pyrazolo[4,3-*d*]pyrimidine derivatives (**17**). The ^1^H-NMR spectrum of compound (**17**) showed the signals at 1.20 (s, 3H, CH_3_), 1.90 (brs, 4H), 3.72 (t, *J* = 6.6 Hz, 2H), 4.54 (t, *J* = 5.6 Hz, 2H), 7.02 (s, 1H), 7.30 (d, 2H, *J* = 8.1 Hz, Ar-H), 7.60 (d, 2H, *J* = 8.2 Hz, Ar-H), 7.70 (m, 1H), 7.78 (m, 1H), 7.91 (m, 1H), 9.30 (br,1H, NH, D_2_O exchangeable) (cf. Scheme 4 and Experimental Section).

Finally, the coupling at the 7-oxo position of compound (**15**) with allyl bromide and bromoethyne give the *O*-alkylation products (**19**) and (**21**), respectively.

### 2.2. Antimicrobial Evaluation

The molar refractivity and the values of the MIC against the tested microorganisms are reported in Table 1, Table 2 and Table 3. Among the 24 newly synthesized compounds, compounds **14b** with MIC value ranging from (6–8 µg·mL^−1^) and **17** with MIC (4–10 µg·mL^−1^) were found to have more potent antibacterial activity against all strains than the reference drug Cefotaxime with MIC value (6–13 µg·mL^−1^). It was found that lipophilicity plays a major role in determining where drugs are distributed within the body after adsorption and, as a consequence, how rapidly they are metabolized and excreted. In the biological system drug disposition depends on the ability to cross membranes, so there is a strong relationship with measures of lipophilicity [23]. So the strong lipophilic character of the molecule plays a major role in producing the antimicrobial effect. In this context the presence of the hydrophobic moiety would be important for such activity. The lipophilicity of the compounds, expressed as log P, explains the main predictor for the activity. The octanol/water partition coefficient C log P was calculated using the software ACD/log P 1.0 and the results are shown in Table 1. The molar refractivity (MR) of the newly synthesized compounds was also calculated using the software ACD/log P 1.0 to explain the activity behavior of the synthesized compounds. From Table 2 and Table 3 it can be inferred that the higher value of molar refractivity favors the activity ratio.

Pyrazole containing *N*-methyl piperazine **7b** with MIC (6–12 µg·mL^−1^) displayed not only comparable antibacterial activity against all the tested bacterial strains, but also excellent antifungal activity against *Aspergillus niger* and *Candida albicans* in low inhibitory concentrations MIC (6 µg·mL^−1^), but moderate activity against *Aspergillus flavus* with MIC (10 µg·mL^−1^). Furthermore compounds **6**, **9**, and **10d**, and pyrazole-containing sulphonamide derivatives **12a**,**b** and **14a** with MIC (8–16 µg·mL^−1^) exhibited good antibacterial activity slightly lower than the reference drug cefotaxime, but **12c**,**d** and **13a**,**b** exhibited moderate antibacterial activity. Also compounds **2**–**4**, **19**, and **21** with MIC (10–16 µg·mL^−1^) exhibited moderate inhibitory efficiency against all the tested bacterial strains.

Antifungal evaluation in vitro revealed that most of the newly prepared compounds exhibited completely different results in comparison with their antibacterial activities. Pyrazoles **2**–**4** with an oxiran-2-yl-methyl group and derivatives of triazolyl moieties at the *N*-1 position showed good inhibitory efficiency against all the tested fungal strains, which gave comparable or superior inhibitory potency to the first-line antifungal drug miconazole. Compound **7b** exhibited equipotent activity against *Aspergillus niger* and *Candida albicans*, but good activity against *Aspergillus flavus* compared to miconazole. Their activity may be attributed to the presence of *N*-butylmethylpiprazine. Compound **12b**, which has sulphonamide derivatives, exhibited equipotent activity against *Aspergillus niger* and *Candida albicans* but good activity against *Aspergillus flavus*; pyrazolopyrimidine sulphonamide derivatives (**13a**) exhibited good antifungal activities with MIC value (8–10 µg·mL^−1^). Most of the newly synthesized compounds (**6**, **7**(**a**,**c**,**d**), **9**, **10**(**a**,**c**,**d**), **12**(**a**,**c**,**d**), **13b**, **17**, **19**, and **21** exhibited moderate antifungal activity against some of the fungal strains, while compounds **10b** and **14a**,**b** exhibited low antifungal activity compared to the reference drug miconazole. 

## 3. Experimental Section

### 3.1. General Information

All melting points were measured on an Electrothermal 9100 series digital melting point apparatus (Shimadzu, Tokyo, Japan). Microanalytical data were gathered with a Vario Elementar apparatus (Shimadzu). Elemental analyses of all compounds were within ±0.4% of the theoretical values. The IR spectra (KBr) were recorded on a Perkin Elmer 1650 spectrometer (Shelton, CT, USA). ^1^H-NMR and ^13^C-NMR spectra were recorded on a JEOL EX-300 and JEOL ECA-500 (Shimadzu, Tokyo, Japan). Chemical shifts were expressed in ppm relative to SiMe_4_ as internal standard in DMSO-*d*_6_ as a solvent. Mass spectra were recorded on a 70 eV Finnigan SSQ 7000 spectrometer (Thermo-Instrument System Incorporation, Columbia, MD, USA). The purity of the compounds was checked on aluminum plates coated with silica gel (Merck, Darmstadt, Germany). Chemicals and solvents (Analar ≥ 99%) were purchased from Sigma-Aldrich (St. Louis, MO, USA).

### 3.2. Synthesis

*Ethyl 4-amino-3-(4-chlorophenyl)-1H-pyrazol-5-carboxylate* (**1**) was achieved by a reported method [19,20,21,22].

*Ethyl 4-amino-3-(4-chlorophenyl)-1-(oxiran-2-ylmethyl)-1H-pyrazole-5-carboxylate* (**2**). A mixture of compound **1** (2.65 g, 10 mmol) and potassium carbonate (1.51 g, 12 mmol) was stirred in 2-(chloromethyl)-oxirane (15 mL) under reflux for 4 h. After the reaction was completed, the mixture was cooled to room temperature, the excess 2-(chloromethyl)-oxirane was evaporated under reduced pressure, and water was added. The residue was extracted with chloroform, the combined organic phase was dried over anhydrous sodium sulfate, and then the residue was purified by column chromatography (eluent, chloroform/methanol 70:1, *v*/*v*) to give the desired compound **2** as a yellow solid, yield: 70%, m.p. 210–212 °C; IR (KBr, cm^−1^): ν 3390 (NH_2_), 3020 (CH-arom.), 2978 (CH-aliph.), 1725(C=O), 1610 (C=N); ^1^H-NMR (500 MHz, DMSO-*d*_6_, δ, ppm); 1.35 (t, 3H, CH_3_), 2.54–2.55 (m, 2H, OCH_2_), 3.22–3.40 (m, 2H, N-CH_2_), 4.25 (q, 2H, OCH_2_CH_3_), 4.85–4.90 (m, 1H, O-CH), 7.25 (d, 2H, *J* = 8.2 Hz, Ar-H), 7.57 (d, 2H, *J* = 8.2 Hz, Ar-H), 7.80 (br, 2H, NH_2_, D_2_O exchangeable); its MS (*m*/*z*), 321 (M^+^); C_15_H_16_ClN_3_O_3_ (321.7); calcd; % C: 55.99, % H: 5.01, % N: 13.06; found; % C: 55.79, % H: 5.00, % N: 13.03.

*Ethyl 4-amino-3-(4-chlorophenyl)-1-[2-hydroxy-3-(1H-1,2,4-triazol-1-yl)-propyl]-1H-pyrazole-5-carboxylate* (**3**) To a stirred suspension of potassium carbonate (1.51 g, 12 mmol) in ethanol was added 1,2,4-triazole (6.91 g, 10 mmol). The mixture was stirred at 60 °C for 1 h. The reaction was cooled to room temperature, compound **2** (3.22 g, 10 mmol) was added at the room temperature and stirred for 2 h under reflux. After the reaction came to an end, the solvent was evaporated and the residue was extracted with chloroform. The combined organic extracts were dried over anhydrous sodium sulfate and concentrated under reduced pressure. The crude product was purified by column chromatography (eluent, chloroform/methanol 30:1, *v*/*v*) to afford compound **3** as a white solid. Yield: 60%; m.p. 250 °C; IR (KBr, cm^−1^): ν 3443 (broad OH, NH_2_), 3016 (CH-arom.), 2989 (CH-aliph.), 1730(C=O), 1615 (C=N), ^1^H-NMR (500 MHz, DMSO-*d*_6_, δ, ppm): 1.26 (t, 3H, CH_3_), 4.19–4.69 (m, 6H, -CH_2_-), 4.97 (m, 1H, O-CH), 5.66 (m, 1H, OH), 7.30 (d, 2H, *J* = 8.4 Hz, Ar-H), 7.62 (d, 2H, *J* = 8.5 Hz, Ar-H), 7.88 (br, 2H, NH_2_, D_2_O exchangeable), 8.07 (s, 1H, triazole 5-H), 8.85 (s, 1H, triazole 3-H); its MS (*m/z*), 390 (M^+^); C_17_H_19_ClN_6_O_3_ (390.8): calcd; % C: 52.24, % H: 4.90, % N: 21.50; found; % C: 52.21, % H: 4.90, % N: 21.48.

*4-Amino-3-(4-chlorophenyl)-1-[2-hydroxy-3-(1H-1,2,4-triazol-1-yl)propyl]-1H-pyrazole-5-carboxylic acid (***4***)*. To a stirred solution of sodium hydroxide (3%, 20 mL) was added compound **3** the mixture was stirred at 100 °C for 3 h. After the reaction was completed, the mixture was treated with formic acid to adjust the pH to 7, and then the suspension was filtered and washed with water three times to give compound **4** as a white solid. Yield: 75%; m.p. 270–272 °C; IR (KBr, cm^−1^): ν 3420 (broad OH, NH_2_), 3020 (CH-arom.), 2992 (CH-aliph.), 1730(C=O), 1618 (C=N), ^1^H-NMR (500 MHz, DMSO-*d*_6_, δ, ppm): 4.36–4.84 (m, 4H, 2CH_2_), 5.05 (m, 1H, O-CH), 5.77 (m, 1H, OH), 7.25 (d, 2H, *J* = 8.0 Hz, Ar-H), 7.60 (d, 2H, *J* = 8.1 Hz, Ar-H), 7.85 (br, 2H, NH_2_, D_2_O exchangeable), 8.07 (s, 1H, triazole 5-H), 8.95 (s, 1H, triazole 3-H), 14.90 (s, 1H, COOH); its MS (*m/z*), 362 (M^+^), C_15_H_15_ClN_6_O_3_ (362.7): calcd: % C: 49.66, % H: 4.17, % N: 23.17; found; % C: 49.65, % H: 4.15, % N: 23.14.

#### 3.2.1. General Procedure for the Synthesis of **6** and **9**

A mixture of compound **1** (2.65 g, 10 mmol) and 1,4-dibromobutane **5** (2.16 g, 10 mmol) and /or (*E*)-1,4-dibromobut-2-ene **8** (2.14 g, 10 mmol) in dry DMF (10 ml) along with anhydrous potassium carbonate (1.88 g, 15 mmol) was stirred at room temperature for about five hours. The reaction mixture was then poured into ice cold water. The contents were then extracted with diethyl ether and the ether layer was washed with a brine solution and dried over anhydrous sodium sulfate. The solvent was removed and the crude product so obtained was crystallized from ethanol. 

*Ethyl 4-amino-1-(4-bromobutyl)-3-(4-chlorophenyl)-1H-pyrazole-5-carboxylate* *(***6***)*. Pale yellow solid. Yield: 75%, m.p. 190–192 °C; IR (KBr, cm^−1^): ν 3390, 3360 (NH_2_), 3018 (CH-arom.), 2983 (CH-aliph.), 1725 (C=O), 1620 (C=N), ^1^H-NMR (500 MHz, DMSO-*d*_6_, δ, ppm): 1.35 (t, 3H, CH_3_), 2.20–2.25 (m, 4H, 2CH_2_), 3.40 (t, 2H, CH_2_), 4.10 (q, 2H, OCH_2_), 4.28 (t, 2H, CH_2_), 7.25 (d, 2H, *J* = 8.2 Hz, Ar-H), 7.57 (d, 2H, *J* = 8.3 Hz, Ar-H), 7.80 (br, 2H, NH_2_, D_2_O exchangeable); its MS (*m/z*), 400 (M^+^), C_16_H_19_BrClN_3_O_2_ (400.6): calcd; % C: 47.96, % H: 4.78, % N: 10.49; found; % C: 47.94, % H: 4.77, % N: 10.47.

*Ethyl 4-amino-1-[(2E)-(4-bromobut-2-en-1-yl)-3-(4-chlorophenyl)-1H-pyrazole-5-carboxylate* *(***9***)*. Pale yellow solid. Yield: 70%, m.p. 196–198 °C; IR (KBr, cm^−1^): ν 3400, 3375 (NH_2_), 3020 (CH-arom.), 2980 (CH-aliph.), 1725 (C=O), 1618 (C=N), 1585 (C=C); ^1^H-NMR (500 MHz, DMSO-*d*_6_, δ, ppm): 1.33 (t, 3H, CH_3_), 3.54 (t, 2H, CH_2_), 4.14 (t, 2H, CH_2_), 4.27 (q, 2H, OCH_2_), 6.09–6.12 (m, 1H, =CH), 6.13–6.19 (m, 1H, =CH), 7.28 (d, 2H, *J* = 8.3 Hz, Ar-H), 7.60 (d, 2H, *J* = 8.4 Hz, Ar-H), 7.90 (br, 2H, NH_2_, D_2_O exchangeable); its MS (*m/z*), 398 (M^+^), C_16_H_17_BrClN_3_O_2_ (398.6): calcd; % C: 48.20, % H: 4.30, % N: 10.54; found; % C: 48.19, % H: 4.28, % N: 10.51.

#### 3.2.2. General Procedure for Synthesis of **7a**–**d** and **10a**–**d**

*N*-Nucleophile (10 mmol) was dissolved in DMF (20 mL) along with anhydrous potassium carbonate (15 mmol) The bromo compound (**6** and **9**) (10 mmol) was added to the solution, and the mixture was stirred at room temperature for 3 h. After completion of the reaction, the mixture was poured into ice cold water. The precipitate obtained was washed with water, dried under a vacuum, and crystallized from ethanol to afford **7a**–**d** and from dioxane to afford **10a**–**d** in good yield.

*Ethyl 4-amino-1-(4-(piperidin-1-yl)butyl))-3-(4-chlorophenyl)-1H-pyrazole-5-carboxylate* *(***7a***)*. White solid, m.p. 160–162 °C; IR (KBr, cm^−1^): ν 3385, 3360 (NH_2_), 3023 (CH-arom.), 2978 (CH-aliph.), 1730 (C=O), 1622 (C=N), ^1^H-NMR (500 MHz, DMSO-*d*_6_, δ, ppm): 1.34 (t, 3H, CH_3_), 1.44–1.55 (m, 2H, piperidine), 1.61–1.63 (m, 4H, piperidine), 1.77–1.82 (m, 2H, piperidine), 2.02 (m, 2H, piperidine), 2.22–2.30 (m, 4H, 2CH_2_), 3.40 (t, 2H, CH_2_), 3.68 (t, 2H, CH_2_), 4.09 (q, 2H, OCH_2_), 7.20 (d, 2H, *J* = 8.1 Hz, Ar-H), 7.57 (d, 2H, *J* = 8.3 Hz, Ar-H), 7.84 (br, 2H, NH_2_, D_2_O exchangeable), ^13^C-NMR (DMSO-*d*_6_) δ 18.28 (CH_3_), 23.86, 24.39, 25.19, 25.44, 25.89, 39.87 (6CH_2_), 42.58 (OCH_2_), 48.15, 48.67 (2 NCH_2_), 117.12, 120.83, 122.37, 124.41, 125.82, 129.92, 130.18, 151.4, 153.07 (nine signals for 9 sp2 carbon), 172.64 ppm (carbonyl) ppm; its MS (*m/z*), 404 (M^+^), C_21_H_29_ClN_4_O_2_ (404.9): calcd; % C: 62.29, % H: 7.22, % N: 13.84; found; % C: 62.29, % H: 7.20, % N: 13.81.

*Ethyl 4-amino-1-(4-(4-methylpiperazin-1-yl)butyl)-3-(4-chlorophenyl)-1H-pyrazole-5-carboxy-late* *(***7b***)*. White solid, m.p. 156–158 °C; IR (KBr, cm^−1^): ν 3395, 3363 (NH_2_), 3020 (CH-arom.), 2982 (CH-aliph.), 1725 (C=O), 1622 (C=N), ^1^H-NMR (500 MHz, DMSO-*d*_6_, δ, ppm), 1.33 (t, 3H, CH_3_), 1.75–1.80 (m, 4H, 2CH_2_), 2.03 (t, *J* = 6.8 Hz, 2H, CH_2_), 2.29 (s, 3H, piperazinyl NCH_3_), 2.59 (brs, 4H, piperazinyl, CH_3_N(CH_2_)_2_), 3.36 (brs, 4H, piperazinyl, N(CH_2_)_2_), 4.02 (t, *J* = 6.0 Hz, 2H, CH_2_), 4.15 (q, 2H, OCH_2_), 7.23 (d, 2H, *J* = 8.0 Hz, Ar-H), 7.58 (d, 2H, *J* = 8.2 Hz, Ar-H), 7.90 (br, 2H, NH_2_, D_2_O exchangeable), its MS (*m/z*), 419 (M^+^), C_21_H_30_ClN_5_O_2_ (419.9): calcd; % C: 60.06, % H: 7.20, % N: 16.68; found; % C: 60.04, % H: 7.20, % N: 16.66.

*Ethyl 4-amino-1-(4-morpholinobutyl)-3-(4-chlorophenyl)-1H-pyrazole-5-carboxylate* *(***7c***).* White solid, m.p. 175–178 °C; IR (KBr, cm^−1^): ν 3400, 3370 (NH_2_), 3022 (CH-arom.), 2978 (CH-aliph.), 1730 (C=O), 1618 (C=N); ^1^H-NMR (500 MHz, DMSO-*d*_6_, δ, ppm); 1.33 (t, 3H, CH_3_), 1.78–1.80 (m, 4H, 2CH_2_), 2.03–2.05 (m, 2H, CH_2_), 2.20 (t, 2H, CH_2_), 3.29 (t, 4H, morpholinyl, N(CH_2_)_2_, *J* = 5.0 Hz), 3.90 (t, 4H, morpholinyl, O(CH_2_)_2_), *J* = 5.0 Hz), 4.05 (t, 2H, CH_2_), 4.15 (q, 2H, OCH_2_), 7.20 (d, 2H, *J* = 8.3 Hz, Ar-H), 7.58 (d, 2H, *J* = 8.4 Hz, Ar-H), 7.89 (br, 2H, NH_2_, D_2_O exchangeable); its MS (*m/z*), 406 (M^+^); C_20_H_27_ClN_4_O_3_ (406.9); calcd; % C: 59.03, % H: 6.69, % N: 13.77; found; % C: 59.02, % H: 6.67, % N: 13.75.

*Ethyl 4-amino-1-(4-(pyrrolidin-1-yl)butyl)-3-(4-chlorophenyl)-1H-pyrazole-5-carboxylate (***7d***).* White solid, m.p. 173–175 °C; IR (KBr, cm^−1^): ν 3410, 3380 (NH_2_), 3022 (CH-arom.), 2978 (CH-aliph.), 1726 (C=O), 1625 (C=N); ^1^H-NMR (500 MHz, DMSO-*d*_6_, δ, ppm); 1.35 (t, 3H, CH_3_), 1.75–1.79 (m, 2H, CH_2_), 2.05–2.08 (m, 2H, CH_2_), 2.20 (t, 2H, CH_2_), 2.53 (brs, 4H, 2CH_2_ pyrrole), 2.76–2.89 (br, 2H, 2CH_2_), 4.09 (t, 2H, CH_2_), 4.22 (q, 2H, OCH_2_), 7.25 (d, 2H, Ar-H), 7.64 (d, 2H, *J* = 8.3 Hz, Ar-H), 7.89 (br, 2H, *J* = 8.5 Hz, NH_2_, D_2_O exchangeable); its MS (*m/z*), 390 (M^+^); C_20_H_27_ClN_4_O_2_ (390.9); calcd; % C: 61.45, % H: 6.69, % N: 14.33; found; % C: 61.43, % H: 6.67, % N: 14.30.

*(E)-Ethyl 4-amino-1-(4-(piperidin-1-yl)but-2-en-1-yl)-3-(4-chlorophenyl)-1H-pyrazole-5-carboxylate* *(***10a***)*. White solid, m.p. 223–225 °C; IR (KBr, cm^−1^): ν 3380 (NH_2_), 3020 (CH-arom.), 2980 (CH-aliph.), 1726 (C=O), 1628 (C=N), 1595(C=C), ^1^H-NMR (500 MHz, DMSO-*d*_6_, δ, ppm): 1.35 (t, 3H, CH_3_), 2.72 (brs, 4H, piperidine), 2.39 (brs, 4H, piperidine), 1.59 (m, 2H, piperidine), 3.04 (d, 2H, CH_2_), 4.15 (d, 2H, CH_2_), 4.30 (q, 2H, OCH_2_), 5.89–6.00 (m, 2H, 2CH), 7.30 (d, 2H, *J* = 8.0 Hz, Ar-H), 7.65 (d, 2H, *J* = 8.2 Hz, Ar-H), 7.93 (br, 2H, NH_2_, D_2_O exchangeable); ^13^C-NMR (DMSO-*d*_6_) δ 19.14 (CH_3_), 24.76, 25.56, 25.95, 40.11 (4CH_2_), 42.58 (OCH_2_), 48.15, 48.67 (2NCH_2_), 118.19, 119.38, 120.87, 123.27, 124.78, 125.90, 129.25, 130.18, 131.21, 151.81, 153.11 (11 signals for 11 sp2 carbon), 173.21 ppm (carbonyl) ppm; its MS (*m/z*), 402 (M^+^); C_21_H_27_ClN_4_O_2_ (402.9): calcd: % C: 62.60, % H: 6.75, % N: 13.91; found; % C: 62.59, % H: 6.73, % N: 13.90.

*(E)-Ethyl-4-amino-1-(4-(4-methylpiperazin-1-yl)but-2-en-1-yl)-3-(4-chlorophenyl)-1H-pyrazole-5-carboxy-late* *(***10b***)*. White solid, m.p. 185–187 °C; IR (KBr, cm^−1^): ν 3387 (NH_2_), 3022 (CH-arom.), 2980 (CH-aliph.), 1727 (C=O), 1630 (C=N), 1590 (C=C); ^1^H-NMR (500 MHz, DMSO-*d*_6_, δ, ppm); 1.30 (t, 3H, CH_3_), 2.03 (d, 2H, CH_2_), 2.20 (s, 3H, -NCH_3_), 2.54 (brs, 4H, piperazinyl, -N(CH_2_)_2_), 3.40 (brs, 4H, piperazinyl, N(CH_2_)_2_), 4.09 (d, 2H, CH_2_), 4.20 (q, 2H, OCH_2_), 5.88–5.90 (m, 1H, CH), 5.97–5.99 (m, 1H, CH), 7.25 (d, 2H, *J* = 8.2 Hz, Ar-H), 7.60 (d, 2H, *J* = 8.2 Hz, Ar-H), 7.90 (br, 2H, NH_2_, D_2_O exchangeable); its MS (*m/z*), 417 (M^+^); C_21_H_28_ClN_5_O_2_ (417.9); calcd; % C: 60.35, % H: 6.75, % N: 16.76; found; % C: 60.32, % H: 6.73, % N: 16.75.

*(E)-Ethyl 4-amino-1-(4-morpholin-1-yl)but-2-en-1-yl)-3-(4-chlorophenyl)-1H-pyrazole-5-carboxylate* *(***10c***)*. White solid, m.p. 215–217 °C; IR (KBr, cm^−1^): ν 3383 (NH_2_), 3028 (CH-arom.), 2978 (CH-aliph.), 1728 (C=O), 1625 (C=N), 1595 (C=C); ^1^H-NMR (500 MHz, DMSO-*d*_6_, δ, ppm); 1.30 (t, 3H, CH_3_), 2.20 (d, 2H, CH_2_), 3.30 (t, 4H, morpholinyl, N(CH_2_)_2_), 3.95 (t, 4H, morpholinyl, O(CH_2_)_2_), 4.05 (d, 2H, CH_2_), 4.20 (q, 2H, OCH_2_), 5.98–6.12 (m, 2H, 2CH), 7.23 (d, 2H, *J* = 8.4 Hz, Ar-H), 7.58 (d, 2H, *J* = 8.5 Hz, Ar-H), 7.89 (br, 2H, NH_2_, D_2_O exchangeable); its MS (*m/z*), 404 (M^+^); C_20_H_25_ClN_4_O_3_ (404.8); calcd; % C: 59.33, % H: 6.22, % N: 13.84; found; % C: 59.32, % H: 6.22, % N: 13.82.

*(E)-Ethyl 4-amino-1-(4-(pyrrolidin-1-yl)but-2-en-1-yl)-3-(4-chlorophenyl)-1H-pyrazole-5-carboxylate* *(***10d***).* White solid, m.p. 180–182 °C; IR (KBr, cm^−1^): ν 3380 (NH_2_), 3022 (CH-arom.), 2978 (CH-aliph.), 1730 (C=O), 1628 (C=N),1590 (C=C); ^1^H-NMR (500 MHz, DMSO-*d*_6_, δ, ppm); 1.29 (t, 3H, CH_3_), 2.25 (d, 2H, CH_2_), 2.55 (brs, 4H, 2CH_2_ pyrrole), 2.69–2.78 (brs, 4H, 2CH_2_ pyrrole), 4.10 (d, 2H, CH_2_), 4.22 (q, 2H, OCH_2_), 5.92–6.12 (m, 2H, 2CH), 7.28 (d, 2H, *J* = 8.2 Hz, Ar-H), 7.66 (d, 2H, *J* = 8.3 Hz, Ar-H), 7.92 (br, 2H, NH_2_, D_2_O exchangeable); its MS (*m/z*), 388 (M^+^); C_20_H_25_ClN_4_O_2_ (388.8); calcd; % C: 61.77, % H: 6.48, % N: 14.41; found; % C: 61. 75, % H: 6. 47, % N: 14. 39.

*Ethyl 3-(4-chlorophenyl)-4-isothiocyanato-1H-pyrazol-5-carboxylate* (**11**). A mixture of compound **1** (2.65 g, 10 mmol) and thiophosgene (1.14 mL, 10 mmol) in dry chloroform (25 mL) was stirred under reflux for 5 h. The solvent was evaporated and the solid obtained was recrystallized from ethanol to give **11** as a deep yellow solid, yield: 70%. m.p. 190–192 °C; IR (KBr, cm^−1^): ν 3386 (NH), 3019 (CH-arom.), 2980 (CH-aliph.), 1728 (C=O), 1604 (C=N), 1265 (C=S); ^1^H-NMR (500 MHz, DMSO-*d*_6_, δ, ppm); 1.35 (t, 3H, CH_3_), 4.20 (q, 2H, OCH_2_), 7.25 (d, 2H, *J* = 8.0 Hz, Ar-H), 7.58 (d, 2H, *J* = 8.1, Hz Ar-H), 9.05 (br, 1H, NH, D_2_O exchangeable); its MS (*m/z*), 307 (M^+^); C_13_H_10_ClN_3_O_2_S (307.7); calcd; % C: 50.73, % H: 3.28, % N: 13.65; found; % C: 50.71, % H: 3.26, % N: 13.62.

#### 3.2.3. General Procedure for Synthesis of **12a**–**d**

A mixture of compound **11** (3.07 g, 10 mmol) and sulfa drugs (10 mmol) in DMF (30 mL) was stirred under reflux for 4 h. The reaction mixture was poured onto ice water and the obtained product was recrystallized from dioxane to give compounds **12a**–**d**, respectively.

*Ethyl-3-(4-chlorophenyl)-3-(4-(N-(3,4*-*dimethyl[1,2]isooxazolo)sulfamoyl)phenyl)thioureido-1H-pyrazole-5-carboxylate (***12a***)*. Pale brown solid, yield: 75%, m.p. 250–252 °C; IR (KBr, cm^−1^): ν 3386, 3350, 3201 (NH), 3019 (CH-arom.), 2989, 2850 (CH-aliph.), 1725 (C=O),, 1612 (C=N), 1260 (C=S), 1325, 1153 (SO_2_); ^1^H-NMR (500 MHz, DMSO-*d*_6_, δ, ppm); 1.29 (t, 3H, CH_3_), 2.20 (s, 3H, CH_3_), 2.28 (s, 3H, CH_3_), 4.18 (q, 2H, OCH_2_), 7.20 (d, 2H, *J* = 8.1 Hz, Ar-H), 7.55 (d, 2H, *J* = 8.2 Hz, Ar-H), 7.88 (d, 2H, *J* = 8.2 Hz, Ar-H), 8.10 (d, 2H, *J* = 8.2 Hz, Ar-H), 9.00 (s, 1H, NH-Ph, exchangeable with D_2_O), 9.15 (br, 2H, 2NH, D_2_O exchangeable), 10.90 (s, 1H, SO_2_NH, exchangeable with D_2_O); its MS (*m/z*), 575 (M^+^); C_24_H_23_ClN_6_O_5_S_2_ (575); calcd; % C: 50.13, % H: 4.03, % N: 14.61; found; % C: 50.11, % H: 4.00, % N: 14.60.

*Ethyl-3-(4-chlorophenyl)-3-(4-(N-(2,6-dimethoxypyrimidin-4-yl)sulfamoyl)phenyl)thioureido-1H-pyrazole-5-carboxylate* *(***12b***)*. Brown solid, yield: 65%, m.p. 279–281 °C; IR (KBr, cm^−1^): ν 3398, 3274, 3178 (NH), 3070 (CH-arom.), 2970, 2860 (CH-aliph.), 1732 (C=O), 1620 (C=N), 1265 (C=S), 1388, 1168 (SO_2_); ^1^H-NMR (500 MHz, DMSO-*d*_6_, δ, ppm); 1.29 (t, 3H, CH_3_), 3.80, 3.90 (2s, 6H, 2OCH_3_), 4.15 (q, 2H, OCH_2_), 6.90 (d, 2H, *J* = 8.0 Hz, Ar-H), 7.20 (d, 2H, *J* = 8.1 Hz, Ar-H), 7.54 (d, 2H, *J* = 8.3 Hz, Ar-H), 7.90 (d, 2H, *J* = 8.3 Hz, Ar-H), 8.60 (s, 1H, CH pyrimidine), 11.10 (s, 3H, 3NH, exchangeable with D_2_O), 12.40 (s, 1H, SO_2_NH, exchangeable with D_2_O); its MS (*m/z*), 618 (M^+^); C_25_H_24_ClN_7_O_6_S_2_ (618.08); calcd; % C: 48.58, % H: 3.91, % N: 15.86; found; % C: 48.55, % H: 3.90, % N: 15.84.

*Ethyl-3-(4-chlorophenyl)-3-(4-(N-(4,6-dimethylypyrimidin-2-yl)sulfamoyl)phenyl)thioureido-1H-pyrazole-5-carboxylate* *(***12c***).* Brown solid, yield: 65%, m.p. 290–292 °C; IR (KBr, cm^−1^): ν 3390, 3251 (NH), 3035 (CH-arom.), 2950, 2865 (CH-aliph.), 1710 (C=O), 1600 (C=N), 1265 (C=S), 1345, 1161 (SO_2_); ^1^H-NMR (500 MHz, DMSO-*d*_6_, δ, ppm); 1.30 (t, 3H, CH_3_), 2.30 (s, 6H, 2CH_3_), 4.10 (q, 2H, OCH_2_), 6.85 (d, 2H, *J* = 8.1 Hz, Ar-H), 7.19 (d, 2H, *J* = 8.3 Hz, Ar-H), 7.50 (d, 2H, *J* = 8.1 Hz, Ar-H), 7.80 (d, 2H, *J* = 8.4 Hz, Ar-H), 8.05 (s, 1H, CH pyrimidine), 9.00 (br, 1H, NH, D_2_O exchangeable), 11.40 (s, 2H, 2NH, exchangeable with D_2_O), 12.10 (s, 1H, SO_2_NH, exchangeable with D_2_O); its MS (*m/z*), 586 (M^+^); C_25_H_24_ClN_7_O_4_S_2_ (586.08); calcd; % C: 51.23, % H: 4.13, % N: 16.73; found; % C: 51.22, % H: 4.11, % N: 16.71.

*Ethyl-3-(4-chlorophenyl)-N-carbamimidoylbenzenesulfonamido-4-thioureido-1H-pyrazole-5-carboxylate* *(***12d***).* Orange solid, yield: 60%, m.p. >300 °C; IR (KBr, cm^−1^): ν 3250, 3125 (NH, NH_2_), 3050 (CH-arom.), 2970, 2850 (CH-aliph.), 1720 (C=O), 1604 (C=N), 1265 (C=S), 1350, 1113 (SO_2_); ^1^H-NMR (500 MHz, DMSO-*d*_6_, δ, ppm); 1.23 (t, 3H, CH_3_), 3.54 (s, 1H, NH, exchangeable with D_2_O), 4.18 (q, 2H, OCH_2_), 6.80 (s, 2H, NH_2_, exchangeable with D_2_O), 7.25 (d, 2H, *J* = 8.2 Hz, Ar-H), 7.49 (d, 2H, *J* = 8.3 Hz, Ar-H), 7.77 (d, 2H, *J* = 8.2 Hz, Ar-H), 7.90 (d, 2H, *J* = 8.2 Hz, Ar-H), 9.05 (br, 1H, NH, D_2_O exchangeable), 11.30 (s, 2H, 2NH thiourea, exchangeable with D_2_O), 13.10 (s, 1H, SO_2_NH, exchangeable with D_2_O); its MS (*m/z*), 522 (M^+^); C_20_H_20_ClN_7_O_4_S_2_ (522.0); calcd; % C: 46.02, % H: 3.86, % N: 18.78; found; % C: 46.00, % H: 3.84, % N: 18.75. 

#### 3.2.4. General Procedure for Synthesis of **13a**,**b**

A mixture of **12a**,**b** (10 mmol) and hydrazine hydrate (80%, 7mL) in butanol (30 mL) was refluxed for 5 h. After cooling, the reaction mixture was poured onto ice water and the solid obtained was recrystallized from dioxane to give **13a**,**b**, respectively.

*4-{[6-Amino-(N-(3,4-dimethyl[1,2]isooxazolo)sulfamoyl)phenyl)amino}-3-(4-chlorophenyl)-7-oxo-6,7-dihydro-1H-pyrazolo[4,3-d]pyrimidine (***13a***).* Yellow solid, yield: 73%, m.p. >300 °C; IR (KBr, cm^−1^): ν 3350, 3201 (NH, NH_2_), 3055 (CH-arom.), 2989, 2850 (CH-aliph.), 1698 (C=O), 1612 (C=N), 1315, 1153 (SO_2_); ^1^H-NMR (500 MHz, DMSO-*d*_6_, δ, ppm); 2.20 (s, 3H, CH_3_), 2.26 (s, 3H, CH_3_), 5.60 (s, 2H, N-NH_2_, exchangeable with D_2_O), 7.00 (d, 2H, *J* = 8.3 Hz, Ar-H), 7.30 (d, 2H, *J* = 8.1 Hz, Ar-H), 7.89 (d, 2H, *J* = 8.2 Hz, Ar-H), 8.10 (d, 2H, *J* = 8.2 Hz, Ar-H), 9.00, 9.15, 10.90 (3br, 3NH, D_2_O exchangeable); ^13^C-NMR (DMSO-*d*_6_) δ 20.47, 21.32 (2 CH_3_), 118.23, 120.81, 121.27, 122.81, 123.29, 125.65, 126.91, 130.55, 135.12, 137.31, 151.80, 153.17, 154.12, 154.28, 155.23, 155.87, 156.31, 156.92, 157.81, 168.21 ppm (20 signals for 20 sp2 carbon); its MS (*m/z*), 526 (M^+^); C_22_H_19_ClN_8_O_4_S (526.9); calcd; % C: 50.14, % H: 3.63, % N: 21.26; found; % C: 50.12, % H: 3.61, % N: 21.23. 

*4-{[6-Amino-(N-(2,6-dimethoxypyrimidin-4-yl)sulfamoyl)phenyl)amino]}-3-(4-chlorophenyl)-7-oxo-6,7-dihydro-1H-pyrazolo[4,3-d]pyrimidine (***13b***)*. Pale yellow solid, yield: 65%, m.p. >300 °C; IR (KBr, cm^−1^): ν 3301, 3205 (NH, NH_2_), 3035 (CH-arom.), 2993, 2889 (CH-aliph.), 1685 (C=O), 1627 (C=N), 1315, 1157 (SO_2_); ^1^H-NMR (500 MHz, DMSO-*d*_6_, δ, ppm); 3.82 (s, 6H, 2OCH_3_), 5.50 (s, 2H, N-NH_2_,exchangeable with D_2_O), 6.90 (d, 2H, *J* = 8.2 Hz, Ar-H), 7.20 (d, 2H, *J* = 8.1 Hz, Ar-H), 7.58 (d, 2H, *J* = 8.3 Hz, Ar-H), 7.90 (d, 2H, *J* = 8.2 Hz, Ar-H), 8.01 (s, 1H, CH pyrimidine), 8.80 (s, 1H, NH, exchangeable with D_2_O), 9.15 (br, 1H, 1NH, D_2_O exchangeable), 10.80 (s, 1H, SO_2_NH, exchangeable with D_2_O); its MS (*m/z*), 569 (M^+^); C_23_H_20_ClN_9_O_5_S (569.9); calcd; % C: 48.47, % H: 3.54, % N: 22.12; found; % C: 48.46, % H: 3.54, % N: 22.10.

#### 3.2.5. General Procedure for Synthesis of **14a**,**b**

A mixture of compound (**13a**,**b**) (10 mmol) and phenyl isothiocyanate (1.35 g, 10 mmol) in ethanol (50 mL) was refluxed for 10 h. The solvent was then concentrated and the residue was recrystallized from ethanol to give **14a**,**b**, respectively.

*N-(3,4-Dimethyl[1,2]isooxazol-5-yl)-4-3-(4-chlorophenyl)-7-oxo-6-[3-(3-phenylthioureido)-6,7-dihydro-1H-pyrazolo[4,3-d]pyrimidine-5-ylamino)benzenesulfonamide (***14a***)*. Brown solid, yield: 60%, m.p. >300 °C; IR (KBr, cm^−1^): ν 3205 (NH), 3035 (CH-arom.), 2927, 2865 (CH-aliph.), 1668 (C=O), 1550 (C=N), 1242 (C=S), 1315, 1168 (SO_2_); ^1^H-NMR (500 MHz, DMSO-*d*_6_, δ, ppm); 2.15 (s, 3H, CH_3_), 2.21 (s, 3H, CH_3_), 6.90–7.02 (m, 5H, Ar-H), 7.23 (d, 2H, *J* = 8.0 Hz, Ar-H), 7.54 (d, 2H, *J* = 8.0 Hz, Ar-H), 7.89 (d, 2H, *J* = 8.2 Hz, Ar-H), 8.11 (d, 2H, *J* = 8.2 Hz, Ar-H), 8.51, 8.62 (2s, 2H, 2NH, exchangeable with D_2_O), 9.15 (s, 1H, NH exchangeable with D_2_O), 9.70 (s, 1H, NH-Ph, exchangeable with D_2_O), 10.72 (s, 1H, SO_2_NH, exchangeable with D_2_O); its MS (*m/z*), 662 (M^+^); C_29_H_24_ClN_9_O_4_S_2_ (662.1); calcd; % C: 52.60, % H: 3.65, % N: 19.04; found; % C: 52.59, % H: 3.63, % N: 19.02.

*N-(2,6-Dimethoxypyrimidin-4-yl)-4-3-(4-chlorophenyl)-7-oxo-6-[3-(3-phenylthioureido)-6,7-dihydro-1H-pyrazolo[4,3-d]pyrimidine-5-ylamino)benzenesulfonamide (***14b***)*. Brown solid, yield: 63%, m.p. >300 °C; IR (KBr, cm^−1^): ν 3210 (NH), 3030 (CH-arom.), 2928, 2865 (CH-aliph.), 1669 (C=O), 1550 (C=N), 1240 (C=S), 1317, 1168 (SO_2_); ^1^H-NMR (500 MHz, DMSO-*d*_6_, δ, ppm); 3.86 (s, 6H, 2OCH_3_), 6.92–7.05 (m, 5H, Ar-H), 7.25 (d, 2H, *J* = 8.3 Hz, Ar-H), 7.54 (d, 2H, *J* = 8.1 Hz, Ar-H), 7.90 (d, 2H, *J* = 8.2 Hz, Ar-H), 8.14 (d, 2H, *J* = 8.2 Hz, Ar-H), 8.19 (s, 1H, CH pyrimidine), 8.50, 8.60 (2s, 2H, 2NH, exchangeable with D_2_O), 9.15 (s, 1H, NH exchangeable with D_2_O), 9.73 (s, 1H, NH, exchangeable with D_2_O), 10.90 (s, 1H, SO_2_NH, exchangeable with D_2_O); its MS (*m/z*), 705 (M^+^); C_30_H_25_ClN_10_O_5_S_2_ (705.1); calcd; % C: 51.10, % H: 3.57, % N: 19.86; found; % C: 51.10, % H: 3. 55, % N: 19. 84.

*3-(4-Chlorophenyl)-5-methyl-1,6-dihydro-7H-pyrazolo[4,3-d]pyrimidin-7-one (***15***)* was achieved by a reported method [9].

*3-(4-Chlorophenyl)-5-methyl-7-oxybutyl)-isoindoline-1,3-dioxo-1,6-dihydro-7H-pyrazolo[4,3-d]pyrimidin (***17***)*. A mixture of **15** (2.60 g, 10 mmol), *N*-(4-bromobutyl)-phthalimide **16** (2.82 g, 10 mmol) and anhydrous K_2_CO_3_ (1.8, 10 mmol) in 5 mL DMF was stirred in a round bottom flask for 6–8 h at room temperature. The reaction mixture was poured onto ice water and the obtained product was recrystallized from dioxane to give compound **17** as colorless solid, yield: 72%, m.p. 290–292 °C; IR (KBr, cm^−1^): ν 3210 (NH), 3020 (CH-arom.), 2922, 2865 (CH-aliph.), 1680, 1668 (C=O), 1550 (C=N), 1390 (CH_3_), 1080 (C-O); ^1^H-NMR (500 MHz, DMSO-*d*_6_, δ, ppm); 1.20 (s, 3H, CH_3_), 1.90 (brs, 4H, 2CH_2_), 3.72 (t, 2H, CH_2_, *J* = 6.6 Hz, 2H), 4.54 (t, *J* = 5.6 Hz, 2H, CH_2_), 7.02 (s, 1H, Ar-H), 7.30 (d, 2H, *J* = 8.1 Hz, Ar-H), 7.60 (d, 2H, *J* = 8.2 Hz, Ar-H), 7.70 (m, 1H-Ar), 7.78 (m, 1H, Ar-H), 7.91 (m, 1H, Ar-H), 9.30 (br, 1H, NH, D_2_O exchangeable); its MS (*m/z*), 461 (M^+^); C_24_H_20_ClN_5_O_3_ (461.9); calcd; % C: 62.41, % H: 4.36, % N: 15.16; found; % C: 62.39, % H: 4.34, % N: 15.15.

#### 3.2.6. General Procedure for Synthesis of **19** and **21**

A mixture of **15** (2.60 g, 10 mmol), was stirred with potassium carbonate (1.38 g, 10 mmol) in dry DMF (20 mL) for 1 h, followed by the addition of the appropriate alkenyl halide (10 mmol) in portions. The reaction mixture was stirred at room temperature overnight, then refluxed for 4 h. After cooling it was then filtered to remove insoluble materials, and the filtrate was poured into ice water to give the crude product as a precipitate, which in turn was filtered off and dried. The crude product obtained in all cases was purified by recrystallization from ethanol.

*3-(4-Chlorophenyl)-5-methyl-7-(prop-2-en-1-yloxy)-1H-pyrazolo[4,3-d]pyrimidine (***19***)*. This compound was prepared from **15** (2.60 g, 10 mmol) and allyl bromide (1.2 g, 10 mmol) was obtained as a white powder, yield: 75%, m.p. 280–282 °C; IR (KBr, cm^−1^): ν 3220 (NH), 3080 (CH-arom.), 2930, 2870 (CH-aliph.), 1640 (C=C), 1550 (C=N), 1380 (CH_3_), 1150 (C-O); ^1^H-NMR (500 MHz, DMSO-*d*_6_, δ, ppm); 1.23 (s, 3H, CH_3_), 4.12 (d, 2H, OCH_2_), 6.08–6.14 (m, 1H, =CH), 6.15–6.20 (m, 1H, CH_2_), 7.28 (d, 2H, *J* = 8.2 Hz, Ar-H), 7.58 (d, 2H, *J* = 8.2 Hz, Ar-H), 9.28 (br, 1H, NH, D_2_O exchangeable); its MS (*m/z*), 300 (M^+^); C_15_H_13_ClN_4_O (300.7); calcd; % C: 59.91, % H: 4.36, % N: 18.63; found; % C: 59.90, % H: 4.34, % N: 18.60.

*3-(4-Chlorophenyl)-7-(ethynyloxy)-5-methyl-1H-pyrazolo[4,3-d]pyrimidine (***21***).* This compound was prepared from **15** (2.60 g, 10 mmol) and bromoethyne (1.05 g, 10 mmol) was obtained as a white powder, yield: 80%, m.p. 268–270 °C; IR (KBr, cm^−1^): ν 3200 (NH), 3085 (CH-arom.), 2922, 2870 (CH-aliph.), 2150 (C≡C), 1600 (Aryl), 1550 (C=N), 1350 (CH_3_), 1153 (C-O); ^1^H-NMR (500 MHz, DMSO-*d*_6_, δ, ppm); 1.28 (s, 3H, CH_3_), 3.68 (s, 1H, CH), 7.30 (d, 2H, *J* = 8.0 Hz, Ar-H), 7.68 (d, 2H, *J* = 8.2 Hz, Ar-H), 9.25 (br, 1H, NH, D_2_O exchangeable); its MS (*m/z*), 284 (M^+^); C_14_H_9_ClN_4_O (284.7); calcd; % C: 59.06, % H: 3.19, % N: 19.68; found; % C: 59.04, % H: 3.17, % N: 19.65.

### 3.3. Antimicrobial Assay

All the newly synthesized compounds **2**–**4**, **6**, **7a**–**d**, **9**, **10a**–**d**, **12a**–**d**, **13a**,**d**, **14a**,**d**, **17**, **19**, and **21** were screened for their in vitro antimicrobial activity to determine the zone of inhibition at 100 µg/mL against three gram-positive bacteria (*Staphylococcus aureus* ATCC 22096, *Bacillus subtilis* ATCC 23441, and *Micrococcus luteus* ATCC 24698), three gram-negative bacteria (*Escherichia coli* MTCC 23443, *Salmonella typhi* ATCC 27733, and *Klebsiella pneumoniae* ATCC 23432) as well as three fungi (*Aspergillus niger* ATCC 21282, *Aspergillus flavus* ATCC 23277, and *Candida albicans* ATCC 24227) using the cup plate method [24,25], where inoculated Muller–Hilton agar for bacteria and sabouraud dextrose agar for fungi was separately poured onto the sterilized petri dishes (25–30 mL in each petri dish). The poured material was allowed to set (30 min) and thereafter the ‘CUPS’ (8 mm diameter) was made by punching into the agar surface with a sterile cork borer and scooping out the punched part of the agar. Into these cups, the test compound solution (0.1 mL) was added with the help of a micro pipette. The plates were incubated at 37 °C for 14 h for bacteria and 30 h for fungi and the results were noted. The test solution was prepared using DMSO as a solvent. The minimum inhibitory concentration (MIC) of all the compounds was also measured by the serial dilution method [26]. Clinically, antimicrobial drugs cefotaxime and miconazole were used as the positive control and DMSO was used as a blank. 

## 4. Conclusions

Several pyrazole and pyrazolopyrimidine derivatives have been synthesized, starting from pyrazole-5-carboxylate, in good yields. The pharmacological study was undertaken to evaluate the effects of substituents on the antibacterial and antifungal activities. Most of the synthesized compounds exhibited good antibacterial activity towards gram-positive and gram-negative bacteria, and some of the synthesized compounds showed good to moderate antifungal activity. Also, the antimicrobial activity of the synthesized compounds increases with increasing log P and molar refractivity.

## Figures and Tables

**Table 1:** Calculated log P and molar refractivity (MR) of newly synthesized compounds.

Compound	Log P	MR
**2**	3.27	80.99
**3**	2.62	99.74
**4**	1.44	90.17
**6**	4.89	94.32
**7a**	5.16	111.21
**7b**	3.23	114.99
**7c**	3.50	107.99
**7d**	4.59	106.61
**9**	4.73	94.32
**10a**	5.07	111.21
**10b**	3.22	114.99
**10c**	3.50	107.99
**10d**	4.51	106.61
**12a**	5.30	144.21
**12b**	5.78	153.01
**12c**	5.03	149.52
**12d**	3.04	129.90
**13a**	2.63	131.61
**13b**	3.10	140.66
**14a**	4.76	172.84
**14b**	5.23	181.90
**17**	4.07	123.34
**19**	2.82	82.78
**21**	2.91	76.25
Cefotaxime	3.03	97.20
Miconazole	5.09	102.57

**Table 2:** Antibacterial activity of newly synthesized compounds.

MIC in µg/mL and Zone of Inhibition in mm						
Compound	Gram-Pos. Bacteria			Gram-Neg. Bacteria Data		
	*Staphylococcus aureus*	*Bacillus subtilis*	*Micrococcus luteus*	*Escherichia coli*	*Salmonella typhi*	*Klebsiella pneumoniae*
**2**	16/19	10/16	12/16	20/15	12/21	14/21
**3**	18/18	12/20	14/20	16/19	10/18	10/21
**4**	16/14	12/14	16/14	16/16	12/16	14/18
**6**	14/18	10/16	12/20	14/15	10/16	10/16
**7a**	12/20	6/18	10/21	13/18	6/20	8/24
**7b**	12/21	6/19	10/22	14/17	6/18	8/16
**7c**	14/18	10/16	12/20	14/15	10/16	10/16
**7d**	16/18	10/19	12/16	16/10	12/10	12/12
**9**	14/18	10/16	12/20	13/18	10/16	10/16
**10a**	12/19	6/18	9/20	12/16	6/19	8/20
**10b**	16/20	12/18	14/16	16/16	12/20	12/18
**10c**	15/16	10/14	12/16	16/15	10/16	10/10
**10d**	10/22	8/22	12/20	10/19	6/21	10/20
**12a**	12/15	10/16	13/14	16/15	12/16	14/15
**12b**	14/15	8/17	12/18	14/15	10/15	10/16
**12c**	20/19	14/16	20/14	18/15	14/16	18/16
**12d**	21/16	20/18	18/12	20/16	13/17	15/15
**13a**	16/18	10/19	12/22	16/20	10/19	14/22
**13b**	14/20	10/20	14/21	16/19	10/19	12/20
**14a**	10/18	8/22	12/16	16/19	8/18	10/21
**14b**	8/23	6/21	6/23	8/20	6/19	6/22
**17**	10/23	4/22	4/20	10/18	6/21	6/20
**19**	18/15	12/16	16/15	18/14	12/14	14/15
**21**	19/15	12/20	19/18	16/16	12/15	12/16
Cefotaxime	12/23	6/22	10/20	13/22	6/24	8/21

Results expressed as Minimal inhibitory concentration (MIC, µg·mL^−1^)/inhibition zone diameter in mm of the newly synthesized compounds.

**Table 3:** Antifungal activity of newly synthesized compounds.

Compound	MIC in µg/mL and Zone of Inhibition (in mm)		
	*Aspergillus niger*	*Aspergillus flavus*	*Candida albicans*
**2**	4/18	6/18	8/20
**3**	7/20	6/22	6/20
**4**	6/21	8/20	5/22
**6**	10/18	12/14	10/12
**7a**	8/18	25/10	10/15
**7b**	6/23	10/12	6/20
**7c**	12/15	12/14	10/18
**7d**	10/19	25/10	12/15
**9**	10/20	10/16	12/16
**10a**	10/18	12/15	12/14
**10b**	10/16	28/10	25/15
**10c**	10/20	12/15	10/18
**10d**	12/16	25/14	12/20
**12a**	10/17	12/14	12/15
**12b**	6/22	8/20	6/20
**12c**	10/15	25/12	10/18
**12d**	12/15	12/15	25/10
**13a**	8/23	8/21	10/20
**13b**	12/14	10/18	10/16
**14a**	10/20	25/14	28/12
**14b**	25/14	12/15	30/10
**17**	14/15	10/15	12/18
**19**	10/16	10/18	12/16
**21**	12/18	30/10	12/15
Miconazole	6/ 22	6/20	6/22
